# Epidemiology of hypertension in Fulani indigenous populations—age, gender and drivers

**DOI:** 10.1186/s41043-017-0112-2

**Published:** 2017-11-10

**Authors:** Clement Kufe Nyuyki, George Ngufor, George Mbeh, Jean Claude Mbanya

**Affiliations:** 1 Medical Research Council/University of the Witwatersrand, Developmental Pathways for Health Research Unit, Department of Paediatrics, Faculty of Health Sciences, University of the Witwatersrand, Johannesburg, South Africa; 2 Health of Populations in Transition (HoPiT) Research Group, Department of Medicine and Specialities, Faculty of Medicine and Biomedical Sciences, The University of Yaoundé 1, Yaoundé, Cameroon

**Keywords:** Epidemiology, Hypertension, Fulani, Blood pressure, Drivers

## Abstract

**Background:**

Hypertension is a public health problem and the main contributor to cardiovascular mortality and morbidity. Little is known about hypertension among the minority, diverse and socially disadvantaged 23–24 million Fulani/Peul populations dispersed in West, Central and East Africa, undergoing a transition from traditional to transitional and modern lifestyle.

This study describes age and gender variations in blood pressure and drivers of hypertension among rural Fulani population of Cameroon.

**Methods:**

We analysed population-based cross-sectional data collected in 2013 by standard methods from 1337 Fulani/Peul aged ≥ 20 years. Hypertension was defined as systolic blood pressure (SBP) ≥ 140 mmHg and/or diastolic blood pressure ≥ 90 mmHg or current use of anti-hypertensive medication. We elucidated the occurrence and drivers of hypertension by chi-square test, Student’s *t* test and univariate and multivariable logistic regression models.

**Results:**

The prevalence of hypertension was 31.1% (men 36.5% and women 28.7%). Systolic and diastolic blood pressure increased with age. Older women suffered more from grades 1, 2 and 3 hypertension than older men. Old age, divorced/separated, never attended school, current/former smoker, family history (FH) of hypertension, diabetic, underweight and substantially increased risk from waist circumference were independently associated with hypertension. Insomnia and had 8–12 children were the only drivers of hypertension among men.

**Conclusion:**

Prevalence of hypertension was high. Awareness and control were low. Hypertension prevalence increased with age and was more prevalent among men than women. Older women experienced severe hypertension more than older men. Culturally embedded interventions are warranted to curb the high burden of hypertension among the Fulani.

**Electronic supplementary material:**

The online version of this article (10.1186/s41043-017-0112-2) contains supplementary material, which is available to authorized users.

## Background

Hypertension prevalence and incidence have trended upwards and are matched by epidemics of obesity and diabetes. High blood pressure represents a leading global health risk and principal cause of disabilities. In Africa, it is responsible for rising death rates from non-communicable diseases (NCDs) among young and active adults [1, 2]. Globally, the prevalence of systolic blood pressure (SBP) ≥ 140 mmHg increased from 17,307 to 20,526 per 100,000 between 1990 and 2015. Within the same period, loss of disability-adjusted life years (DALY) associated with SBP ≥ 140 mmHg increased from 5.2 million to 7.8 million while the largest numbers of SBP-related deaths were caused by ischaemic heart disease (4.9 million), haemorrhagic stroke (2.0 million) and ischaemic stroke (1.5 million). In 2015, about 874 million adults had SBP ≥ 140 mmHg [2].

Sub-Saharan Africa (SSA) is witnessing a rapid increase in urban populations correlated by the adoption of Western lifestyles with a disproportionate increase in the prevalence of hypertension, a common driver of the epidemic of NCDs. Between 2000 and 2013, hypertension prevalence was estimated at 30% in SSA and ranged from 15 to 70%, varying by mean ages: 30 years (16%), 40 years (26%), 50 years (35%) and 60 years (44%) [3]. In Cameroon, the prevalence of hypertension was 24.6% in 2006 witnessing an almost twofold increase (47.5%) in 2012 [3]. Urban areas had an age-standardised prevalence of 29.7% and awareness of 14.1% [4] with a rural prevalence of 31.1 and 29% awareness in 2013 [5]. Studies on hypertension in SSA hardly focussed on the Fulani/Peul who are a diverse minority group [6] spread in 20 countries in a wide swath of West, Central and East Africa with an estimated population of 7 to 8 million nomadic Fulani (indigenous Mbororo) and 16 million settled Fulani (Fulbe). The Fulani are transitioning from traditional to transitory and modern lifestyles. Their access to healthcare is limited by barriers of language, location and culture [7]. As a result, they disproportionately experience poorer health than most mainstream ethnic groups. Studies in Cameroon highlight the existence of the hypertension pandemic and consider the population as ethnically homogenous though the incidence, prevalence and risk factors of the disease vary by ethnicity [8]. The current data of hypertension exhibit gender variations which are age-dependent [9]. Extant health data on Fulani populations is paltry and little is known about the occurrence, distribution and drivers of hypertension amongst Fulani populations though we have indicated that transition from a nomadic pastoral life to a settled life may be accompanied by an increase in the prevalence of risk factors of NCDs [10]. Studies from different populations reported association of different anthropometric indicators with hypertension. Waist circumference (WC) is associated with morbidity and mortality. Body mass index (BMI) mostly used in clinical settings is a simple and convenient though poor measure of body fat distribution [11, 12]. In order to develop culturally embedded interventions amongst the Fulani indigenous populations aimed at addressing NCDs in general and cardiovascular diseases (CVDs) in particular, we examined the epidemiology of hypertension in Fulani/Peul populations with a focus on age and gender variations using central obesity proxy of WC and BMI.

## Methods

### Study population

This study obtained data from a cross-sectional survey in five localities inhabited for the most part by Fulani in East and Adamawa regions of Cameroon from January to February of 2013. Prior to the study, a census was conducted. A total of 1337 Fulani or Peul aged ≥ 20 years from 555 households were recorded with complete data and included in this analysis. The details of the population are in our previous paper [10].

### Sampling design

A multistage cluster sampling method was used with the five sites constituting the strata [10].

### Procedure

Details of data collection with structured questionnaires are described elsewhere [10]. The questionnaire also assessed alcohol intake, tobacco consumption, salt and sugar intake, fruit and vegetable consumption and sleep. Participants were asked whether they have ever drank alcohol and if yes, the number of standard bottles or any locally produced alcoholic drink (units per day) and whether they drank daily. Participants were asked whether they have ever smoked any tobacco products like cigarettes, cigars, pipes and hand-rolled cigarettes, and if yes, whether on a daily basis? Also, whether they used to smoke in the past, how many years they stopped smoking and whether they used snuff or chewing tobacco. To assess their level of salt and sugar consumption, participants were asked to respond by yes or no to whether they frequently added salt or sugar to food or tea/coffee even when other household members thought the salt or sugar was okay. Fruit and vegetable intake was assessed by requesting the number of days participants consumed these food items per week. Data on sleep was collected by requesting participants to state whether they were satisfied with the sleep they had (normal sleep) or had sleep disturbances and not satisfied with the sleep quality (insomnia) in the past 4 weeks.

Blood pressure was measured thrice by standard methods after a participant had been seated for 5 minutes by use of fully automated calibrated Omron M3 machine at 5 minutes interval. The mean blood pressure value from the two highest values was used.

### Classification of measures

BMI was classified according to WHO criteria. Gender-specific central obesity was defined and classified according to WHO guidelines, and WC was used as a surrogate for central obesity [11, 12].

Hypertension defined as SBP ≥ 140 mmHG and/or DBP ≥ 90 mmHg or current use of anti-hypertensive medication. Blood pressure category was defined by the highest level of blood pressure, whether systolic or diastolic and categorised according to the European Society of Hypertension (ESH) and European Society of Cardiology (ESC). Optimal systolic blood pressure (SBP) was < 120 mmHg and diastolic blood pressure (DBP) < 80 mmHg, normal (SBP 120–129 mmHg and DBP 80–84 mmHg), high normal (SBP 130–139 mmHg and DBP 85–89 mmHg), grade 1 hypertension (SBP 140–159 mmHg and DBP 90–99 mmHg), grade 2 hypertension (SBP 160–179 mmHg and DBP 100–109 mmHg), grade 3 (SBP ≥ 180 mmHg and DBP ≥ 110 mmHg), isolated systolic hypertension (≥ 140) and diastolic (< 90). Isolated systolic hypertension was further graded as 1, 2 or 3 [13]. Proportion of hypertensive participants who reported having been previously diagnosed by a health professional was considered to be aware of hypertension, and participants on treatment was based on those who reported taking anti-hypertensive medication within the past 2 weeks.

Physical activity was classified as low, moderate and vigorous depending on the intensity, duration and frequency of physical activity in occupational and leisure times from the Global Physical Activity instrument included in the questionnaire [14, 15].

Classification of alcohol consumption was into abstainers (never consumed) and occasional (drank five (men), four (women) or more of standard bottles in a single day in the past 12 months) or daily drinkers of standard bottles.

We considered different forms of tobacco consumed (manufactured or hand-rolled cigarettes, cigars or smoked, chewed or inhaled) and classified participants as abstainers (never smoked), current (smoke but not every day) and daily smokers (smoke every day) [16].

Fruit and vegetable intake was based on the frequency of intake per week. From zero to two times a week intake was classified as low, three to five times a week as moderate and six to seven times a week was considered as high [17].

### Data management and statistical analysis

Data was captured with Epi Data 3.0 and analysed using STATA 13.1 SE (StataCorp.2012. College Station, TX: StataCorp LP). Data was weighted, adjusted and computed within 95% confidence intervals (CIs) and at significance of *p* < 0.05. The mean for all continuous variables was computed. We took into consideration the complex sampling design by use of survey commands in the analysis. Study participants’ socio-demographic and lifestyle risk factors were presented descriptively using chi-squared and Student’s *t* test to characterise outcome variable (hypertension) and covariates. We examined the relationship between hypertension and covariates by univariate logistic regression. In order to determine drivers of hypertension, we carried out multivariable logistic analysis in a model with all significant (*p* < 0.05) factors in the univariate analysis adjusted for site and gender and further examined gender-specific drivers in two multivariable logistic regressions adjusted for site. Factors were retained by backward selection. Model assumptions were checked, and variables were checked for collinearity and correlation.

### Ethical approval

Ethical approval was obtained from the National Ethics Committee of Cameroon. Signed or thumb-printed consent was obtained from each participant prior to inclusion in the study. Data was kept confidential, codified and analysed anonymously.

### Role of the funding partner

The sponsor of the project did not play any role in the study design, data collection, analysis and interpretation or writing of this article.

## Results

### Descriptive results

**Fig. 1:**
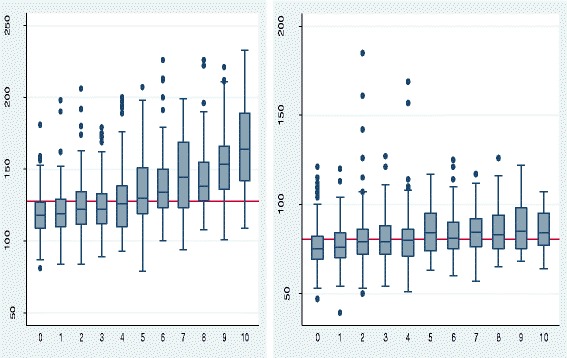
Variation of mean SBP (127.9 mmHg) and DBP (80.4 mmHg) by 5 years age group for Fulani men and women. Age groups: 0 = 20–24, 1 = 25–29, 2 = 30–34, 3 = 35–39, 4 = 40–44, 5 = 45–49, 6 = 50–54, 7 = 55–59, 8 = 60–64, 9 = 65–69 and 10 = ≥ 70 years

**Fig. 2:**
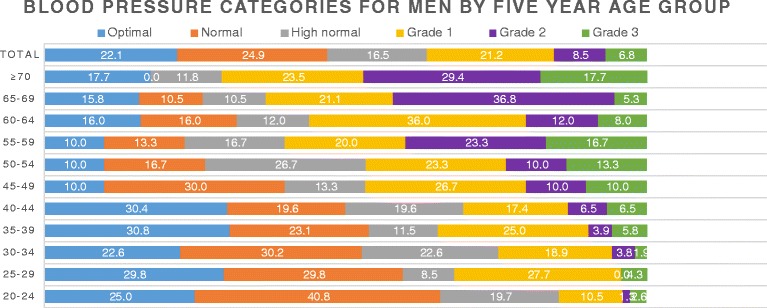
Blood pressure categories for Fulani men aged ≥ 20 years, based on 2013 ESH/ESC guidelines

**Fig. 3:**
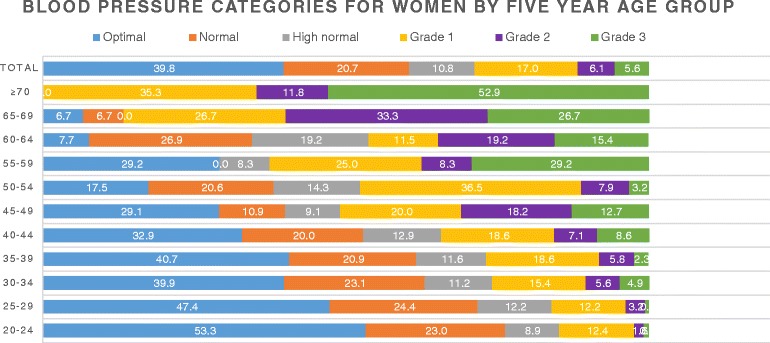
Blood pressure categories for Fulani women aged ≥ 20 years, based on 2013 ESH/ESC guidelines

A total of 1337 consented participants were retained in this analysis, the majority (65.1%) were aged 20–39 years and mostly made of women (68.2%). The mean age of the participants was 36.1 (SD ± 14.4) years, men: 40.2 (SD ± 15.3) years, women: 33.9 (SD ± 13.2) years. The majority (83.7%) of Fulani families did not plan to leave their present site, 7.6% were planning to go elsewhere as against 22.4% for the Bantus around the same area and 8.7% of the Fulani were undecided as to whether they will depart from the present site. Table 1 represents characteristics by prevalence of hypertension and Table 2 univariate and multivariate logistic regression for Fulani men and women aged ≥20 years, and Table 2 represents the correlation of hypertension with socio-demographic and health characteristics. Gender burdens of SBP and DBP by 5-year age interval are shown in Figs. 1, 2 and 3.

**Table 1:** Characteristics by prevalence of hypertension

Factors	Total, 1337	Hypertensive		*p* value
		No, 920 (68.8)	Yes, 417 (31.2%)	
Age group				
20–39	870 (65.1)	690 (79.3)	180 (20.7)	
40–59	348 (26.0)	191 (54.9)	157 (45.1)	
≥ 60	119 (8.9)	39 (32.8)	80 (67.2)	< 0.001
Marital status				
Single	99 (7.4)	78 (78.8)	21 (21.2)	
Presently married	1097 (82.0)	773 (70.5)	324 (29.5)	
Divorced/separated	141 (10.6)	69 (48.9)	72 (51.1)	< 0.001
Education level				
Attended school	313 (23.4)	238 (76.0)	75 (24.0)	
Never	1024 (76.6)	682 (66.6)	342 (33.4)	0.002
Currently employed				
No	1145 (85.6)	791 (69.1)	354 (30.9)	
Yes	192 (14.4)	129 (67.2)	63 (32.8)	0.600
Smoking habit				
Abstainer	1241 (92.8)	872 (70.3)	369 (29.7)	
Former/current smoker	96 (7.2)	48 (50.0)	48 (50.0)	< 0.001
Alcohol drinking				
Never	1249 (93.4)	869 (69.6)	380 (30.4)	
Occasional/daily drinkers	88 (6.6)	51 (57.9)	37 (42.1)	0.023
Vegetable intake				
6–7 days/week	511 (38.2)	355 (69.5)	156 (30.5)	
3–5 days/week	638 (47.7)	436 (68.3)	202 (31.7)	
0–2 days/week	188 (14.1)	129 (68.6)	59 (31.4)	0.917
Fruit intake				
6–7 days/week	188 (14.1)	137 (72.9)	51 (27.1)	
3–5 days/week	391 (29.2)	269 (68.8)	122 (31.2)	
0–2 days/week	758 (56.7)	514 (67.8)	244 (32.2)	0.407
Physical activity				
Vigorous	547 (40.9)	375 (68.6)	172 (31.4)	
Moderate	384 (28.7)	277 (72.1)	107 (27.9)	
Low	406 (30.4)	268 (66.0)	138 (34.0)	0.176
10 min leisure PA				
Vigorous	220 (16.5)	145 (65.9)	75 (34.1)	
Moderate	45 (3.4)	33 (73.3)	12 (26.7)	
Low	1072 (80.1)	742 (69.2)	330 (30.8)	0.503
Frequently added salt to food				
No	1098 (82.1)	749 (68.2)	349 (31.8)	
Yes	239 (17.9)	171 (71.6)	68 (28.4)	0.313
Frequently added sugar to tea/coffee				
No	1103 (82.5)	757 (68.6)	346 (31.4)	
Yes	234 (17.5)	163 (69.7)	71 (30.3)	0.758
FH of hypertension				
No	1141 (85.3)	805 (70.6)	336 (29.4)	
Yes	196 (14.7)	115 (58.7)	81 (41.3)	0.001
FH of diabetes				
No	1225 (91.6)	851 (69.5)	374 (30.5)	
Yes	112 (8.4)	69 (61.6)	43 (38.4)	0.086
FH of obesity				
No	1284 (96.0)	887 (69.1)	397 (30.9)	
Yes	53 (4.0)	33 (62.3)	20 (37.7)	0.294
Diabetes				
No	1283 (96.0)	899 (70.1)	384 (29.9)	
Yes	54 (4.0)	21 (38.9)	33 (61.1)	<0.001
Sleeping difficulties?				
Sleep normally	1033 (77.3)	730 (70.7)	303 (29.3)	
Have insomnia	304 (22.7)	190 (62.5)	114 (37.5)	0.007
Planning to migrate?				
Yes	102 (7.6)	73 (71.6)	29 (28.4)	
No	1119 (83.7)	770 (68.8)	349 (31.2)	
Do not know	116 (8.7)	77 (66.4)	39 (33.6)	0.711
Number of children				
None	91 (6.8)	69 (75.8)	22 (24.2)	
1–2 children	312 (23.3)	237 (76.0)	75 (24.0)	
3–4 children	303 (22.7)	223 (73.6)	80 (26.4)	
5–7 children	293 (21.9)	198 (67.6)	95 (32.4)	
8–12 children	168 (12.6)	81 (48.2)	87 (51.8)	
≥ 13 children	170 (12.7)	112 (65.9)	58 (34.1)	< 0.001
BMI				
Normal weight	607 (45.4)	405 (66.7)	202 (33.3)	
Under weight	622 (46.5)	454 (73.0)	168 (27.0)	
Overweight	78 (5.8)	46 (59.0)	32 (41.0)	
Obese	30 (2.2)	15 (50.0)	15 (50.0)	0.002
WC				
LR (≤ 94 M or ≤ 80 W) cm	1044 (78.1)	734 (70.3)	310 (29.7)	
IR (94 > and < 102, 80 > and < 88) cm	164 (12.3)	113 (68.9)	51 (31.1)	
SIR (≥ 102 M, ≥ 88 W) cm	129 (9.6)	73 (56.6)	56 (43.4)	0.007
BMI, (mean ± SD)	19.5 ± 4.1	19.2 ± 3.9	20.2 ± 4.5	< 0.0001^1^
WC, (mean ± SD)	75.1 ± 11.3	73.9 ± 10.8	77.6 ± 11.9	< 0.0001^1^

^1^ *p*-value from Student’s *t* test

**Table 2:** Univariate and multivariable logistic regression for Fulani men and women aged ≥ 20 years

	Univariate analysis, 1337			Multivariable analysis, 1337		
Factors	OR	95% CI	*p* value	AOR	95% CI	*p* value
Site	1.03	0.93–1.15	0.587	1.05	0.93–1.19	0.450
Gender						
Women	**Ref**			Ref		
Men	**1.42**	**1.12–1.81**	**0.004**	1.13	0.83–1.54	0.83
Age group						
20–39	**Ref**			**Ref**		
40–59	**3.15**	**2.41–4.13**	**< 0.001**	**2.41**	**1.75–3.31**	**< 0.001**
≥ 60	**7.86**	**5.25–11.77**	**< 0.001**	**5.38**	**3.38–8.57**	**< 0.001**
Marital status						
Single	**Ref**			Ref		
Presently married	1.56	0.95–2.54	0.077	1.30	0.72–2.36	0.379
Divorced/separated	**3.87**	**2.16–6.95**	**< 0.001**	***2.04***	*0.99–4.23*	*0.054*
Education level						
Attended school	**Ref**			**Ref**		
Never	**1.59**	**1.19–2.11**	**0.001**	**1.45**	**1.04–2.02**	**0.029**
Smoking habit						
Abstainer	**Ref**			**Ref**		
Former/current smoker	**2.36**	**1.58–3.54**	**< 0.001**	**1.68**	**1.00–2.83**	**0.050**
Alcohol drinking						
Never	**Ref**			Ref		
Occasional/daily drinkers	**1.66**	**1.08–2.55**	**0.021**	1.33	0.75–2.36	0.333
FH of hypertension						
No	**Ref**			**Ref**		
Yes	**1.69**	**1.25–2.29**	**0.001**	**1.65**	**1.18–2.29**	**0.004**
Diabetes						
No	**Ref**			**Ref**		
Yes	**3.68**	**2.07–6.53**	**< 0.001**	**2.92**	**1.56–5.46**	**0.001**
Sleeping difficulties?						
Sleep normally	**Ref**			Ref		
Have insomnia	**1.44**	**1.09–1.91**	**0.010**	1.29	0.95–1.77	0.097
Number of children						
None	**Ref**			Ref		
1–2 children	0.99	0.58–1.69	0.978	1.09	0.58–2.02	0.792
3–4 children	1.13	0.66–1.92	0.664	0.88	0.48–1.63	0.687
5–7 children	1.50	0.88–2.56	0.132	0.95	0.51–1.78	0.870
8–12 children	**3.37**	**1.95–5.83**	**< 0.001**	1.43	0.74–2.79	0.289
≥ 13 children	1.62	0.93–2.84	0.089	1.29	0.69–2.39	0.415
BMI						
Normal weight	**Ref**			**Ref**		
Under weight	**0.74**	**0.57–0.96**	**0.023**	*0.76*	*0.57–1.01*	*0.057* ^1^
Overweight	1.39	0.84–2.32	0.199	1.15	0.67–1.98	0.604
Obese	2.00	0.96–4.17	0.062	1.73	0.84–3.59	0.138
WC						
LR (≤ 94 M or ≤ 80 W) cm	**Ref**			**Ref**		
IR (94 > & < 102 M or 80 > & < 88 W) cm	1.07	0.73–1.56	0.732	0.91	0.61–1.34	0.623^2^
SIR (≥ 102 M or ≥ 88 W)cm	**1.82**	**1.23–2.67**	**0.003**	**1.61**	**1.07–2.42**	**0.022**

^1^Model includes statistically significant variables in univariate analysis and BMI

^2^Model includes statistically significant variables in univariate analysis and WC

Bold implies statistically significant

Italics implies moderate statistical significance

### Overweight and obesity

Overweight and obesity were rare though more prevalent amongst women than men. Significant (*p* < 0.001) differences in the BMI and WC subgroups were observed. No significant gender differences in mean BMI (*p* = 0.2162) but significant gender differences in mean WC (*p* = 0.0005). The prevalence of overweight was 3.5% in men vs. 6.9% in women, and obesity was 1.6% in men against 2.5% in women while the prevalence of increased risk (IR) from WC was 3.8% in men vs. 16.2% in women and Substantially Increaased Risk (SIR) 1.6% in men against 13.4% in women. Men had higher mean BMI than women, and the majority of women of (48.9 vs. 41.4% men) were underweight.

### Systolic and diastolic blood pressure

Mean SBP and mean DBP were 127.9 and 80.4 mmHg, respectively, but higher in men (132.8 and 82.2 mmHg) than women (125.7 and 79.6 mmHg). SBP and DBP increased with advancing age in men and women, although higher mean SBP and DBP values were observed in young men as compared to young women. A sharp decrease in SBP was noted at 60–64 years followed by a hike from ≥ 65 years in women. The general prevalence of hypertension was 31.2%, higher among Fulani men than women.

### Severity of hypertension

A total of 131 (9.8%) participants had isolated systolic hypertension and were graded into 1, 2 or 3 and categorised as optimal (34.2%), normal (22.1%), high normal (12.6%), grade 1 hypertension (18.3%), grade 2 hypertension (6.9%) and grade 3 hypertension (5.9%). Detailed distribution into categories: the prevalence of hypertension among men was 36.5% (optimal (22.1%), normal (24.9%), high normal (16.5%), grade 1 hypertension (21.2%), grade 2 hypertension (8.5%) and grade 3 hypertension (6.8%)) and the prevalence of hypertension among women was 28.7% (optimal (39.8%), normal (20.7%), high normal (10.8%), grade 1 hypertension (17.0%), grade 2 hypertension (6.1%) and grade 3 hypertension (5.6%)). Prevalence of hypertension among the Fulbe was 32% and among the Mbororo 30.8%. Grades 1, 2 and 3 hypertension were higher among the Fulbe than the Mbororo and affecting participants aged ≥ 55 years more than the younger ones (Table 1). Younger women were more likely to have normal blood pressure than younger men, while women aged ≥ 60 years were more likely to have elevated blood pressure than men of the same age group (Figs. 2 and 3).

### Awareness, treatment and control

Of the 417 hypertensive participants, 357 (85.6%) were newly diagnosed, 10 (2.4%) were on anti-hypertensive medication in the past 2 weeks given by health professionals, 9 had uncontrolled blood pressure and 20 (4.8%) had seen a traditional healer for elevated blood pressure in the past 12 months with 11 (2.6%) taking traditional/herbal remedy for high blood pressure at the time of study.

The majority of hypertensive participants were ≥ 40 years old (56.8%), married (77.7%), never went to school (82%), were currently unemployed (84.9%), carried out low physical activity (PA) at leisure (79.1%), carried out vigorous PA (41.2%), had 5–12 children (43.6%), consumed fruits 0–2 days/week (58.5%), consumed vegetables moderately (48.4%) and were underweight (48.4%).

### Co-morbidity and clustering of risk

Also, 88.5% of the non-smokers, 91.1% of the non-drinkers of alcohol, 83.7% of the respondents who did not frequently add salt to food and 82.9% of those who did not always frequently add sugar to tea/coffee, 80.6% of those without family history (FH) of hypertension, 89.7% without FH of diabetes and 95.2% without FH of obesity, 92.1% of participants who had diabetes, 72.7% who slept normally and 74.3% with low risk (LR) from WC were hypertensive. Hypertensive participants had higher BMI and WC when compared to non-hypertensive participants (*p* < 0.0001). Older participants had elevated blood pressure more than younger ones (*p* < 0.001).

### Inferential results

Risk factors associated with hypertension varied by gender in univariate results. Statistically significant factors for men only were: presently married, had insomnia, had ≥ 5 children and increased risk (IR) from WC. Significant factors in univariate analysis for women only: former/current smoker, FH of hypertension, had 8–12 children and overweight. For both men and women, significant factors included: male gender, old age, divorced/separated, never attended school, former/current smoker, occasional/daily drinkers, FH of hypertension, diabetic, had insomnia, had 8–12 children, underweight and SIR from WC.

Adding salt to food, adding sugar to tea/coffee, FH of diabetes, FH of obesity, vegetable and fruit intake, currently employed, physical activity and planning to migrate were not significant in univariate analysis in men and women or both analysed together.

#### Multivariable logistic regression model

All statistically significant factors (*p* < 0.05) in univariate analysis were included in the model adjusted for site and gender with WC replacing BMI to assess the effect of abdominal obesity. Further analysis by gender showed statistically significant results in women for FH of hypertension and in men insomnia.

#### Results of multivariable logistic regression

Old age, never attended school, current/former smoker, FH of hypertension, diabetic and SIR from WC had higher odds were statistically significant. Also divorced/separated and underweight (protective) had moderate statistical significance. Further analysis in men revealed having 8–12 children (moderate) and insomnia to be statistically significant.

Gender was not statistically significant irrespective of whether WC or BMI was considered (Additional files 1 and 2). No association was observed between hypertension and BMI among Fulani women and with marital status in the separate multivariable regression analysis for men and women.

## Discussion

There is a paucity of knowledge on the epidemiology of hypertension amongst the Fulani population. This study provides such data. The epidemiology of hypertension in the Fulani populations should be evolving as a consequence of their progressive shift from traditional lifestyles to transitional and western diet patterns, sedentary life and movement to urban centres.

The mean BMI for both men and women was lower than the estimates from global statistics for Cameroon (men (23.8 Kg/m^2^) and women (25.1 Kg/m^2)^) and was lower than the recommended interval of 21–23 Kg/m^2^ for populations striving for optimal health [18]. The Fulani had a poor diet mainly made of carbohydrates, sweetened beverages and sugar-laden teas and worked for long hours in fields to feed numerous mouths having lost cattle their traditional means of livelihood. This might be an indication of susceptibility to deleterious health, especially among the married Fulani women at reproductive age.

We observed a higher prevalence of SBP, DBP and hypertension among men than women contrary to a study in SSA where women (33.3%) had a higher prevalence than men (31%) [19] and an increase in blood pressure with age as in the Suriname Health Study and in SSA [20, 19]. The prevalence of hypertension among the Fulani (31.2%; men (31.1%) and women (28.7%)) in this study was in the range of the native indigenous community of central Brazil (29.5%) [21], urban dwellers in Cameroon (29.7%, standardised) [4], rural Cameroonians (31.1%) [5] and SSA [19] but lower than that among the aboriginal Nicobarese tribe of India (Car Nicobar island) (50.5%) in 2009 [22]. Unlike urban dwellers in Cameroon whose awareness of hypertension was higher (14.1%) [4] and rural Cameroonians (29%) [5], this young rural Fulani population had a low awareness (2.4%) of hypertension with no significant gender differences and unlike in Brazil with significant gender differences [21]. Prevalence of hypertension in this rural population was high when compared to rural Kenyans with a prevalence of 11.9% among men and 6.3% among women. Though gender prevalence was observed, a twofold gender difference in prevalence was not seen as in rural Kenya [23]. Rural settings and lifestyle have been cited as a protection factor to explain the low prevalence of cardiovascular risk and disease in African populations but the Fulani population, as well as the Maasai in Tanzania, showed a high prevalence of hypertension amongst rural dwellers which may not confer the protective possibility. We have reported an increase in the prevalence of NCD risk factors as a nomadic pastoral Fulani transition to settled life as well as differences in anthropometric and lifestyle factors by ethnicity [10]. The Kenyan Luo Migration Study also showed an increase in blood pressure following rural-urban migration [23]. This high prevalence of grades 1, 2 and 3 hypertension among the young Fulani population with low awareness underpinned by an under-resourced healthcare system may lead to future complications from hypertension, disability, morbidity, mortality and a further increase in prevalence as this population changes from traditional nomadic lifestyle to settled life in urban conglomerates.

The prevalence of hypertension was higher among diabetic, older participants, as well as those who had never attended school, or had a FH of hypertension as in Brazil [21].

We observed associations of hypertension with age, male gender, low level of education, alcohol intake and overweight in univariate analysis as indicated by some studies [22] and with age, lower levels of education, tobacco use in multivariable logistic regression as in SSA including rural Cameroon [5, 23, 24]. Contrary to rural Cameroon [5], being married was not a risk factor of hypertension among the Fulani. Ageing comes with re-distribution of body fat and hormonal changes in men and women and is associated with changes in lifestyle and dietary patterns [25, 26]. This may explain the high prevalence of grades 1, 2 and 3 hypertension with age. The increase in blood pressure in women beyond 65 may be due to menopause which is accompanied by an increase in visceral adipose tissue, decline in oestrogen and testosterone levels and higher circulating inflammatory profile [27–29]. The ageing process also influences testosterone levels which have been shown to have anti-inflammatory actions [30]. At menopause, the cardio-circulatory system is disrupted due to an end to ovarian function. This often leads to a considerable decrease in endothelium-dependent vasodilation and in the emergence of other atherogenic factors. Oestrogen deficiency results in menopause, associated with higher blood pressure and CVD risk in elderly women compared with the premenopausal stages [31, 32]. A decrease in oestrogen concentrations and an increase in BMI may account for older women having inadequate control of blood pressure [33].

Hypertension has been shown to be the cause of some common complications during pregnancy like chronic hypertension, preeclampsia-eclampsia, gestational hypertension and postpartum hypertension which may give rise to maternal and perinatal complications [34]. The majority of women in the study were at the reproductive age, and the aforementioned complications during pregnancy may affect these women. An upsurge in pregnancy-related complications with underlying cause being hypertensive among the Fulani women may be observed in the near future.

Variation in the risk factors of hypertension with body fat proxies (BMI and WC) may be explained by the rarity of BMI defined overweight/obesity and existence of SIR from WC (central obesity) which was not uncommon among the Fulani. Contrary to rural Cameroon [5], BMI-defined obesity was not a risk factor of hypertension among the Fulani but BMI-defined underweight was moderately protective to hypertension in this study. Other studies showed that high BMI is a risk factor for hypertension but we observed an association of high WC with hypertension as observed elsewhere [35, 36]. The values for BMI-defined overweight and obesity were too small in our study to show significant differences at 0.05 level.

Other studies have shown differences in the pathophysiology and life course paths (from foetal life, infancy and childhood to adulthood), gene interaction with the environment, contextual determinants, social gradients [37, 38] and risk factors. Such research is warranted in the Fulani population that is in the transitional phase.

The odds of being hypertensive from central obesity due to visceral adiposity were higher. Visceral adiposity assessed by WC is metabolically more active than peripheral fat responsible for BMI. Ethnicity, genetics, gender and age account for visceral adipose accumulation [39]. Elevated central obesity (visceral adiposity) coupled with high prevalence of hypertension contributes to insulin resistance. The Fulani may have a higher risk of metabolic syndrome increasing the likelihood of type 2 diabetes. High abdominal adiposity and low BMI play a key role in the susceptibility of South Asians to metabolic syndrome [40]. BMI assumes evenly distributed adipose tissue [39] and disregards heterogeneity in regional body fat disposition [41]. The relationship between BMI and morbidity/mortality is curvilinear (J-shaped). Very low BMI (underweight) is associated with increased mortality and a surrogate for morbid conditions [42–44]. However, the relationship between hypertension and BMI was linear in this study. High mortality was observed amongst the Mbororo (14.7%) population [10] which at the same time had a higher prevalence (46.5%) of underweight (age-standardised values, men (31.1%), women (34%)) [10].

In men, insomnia was associated to hypertension as reported elsewhere [45], and having 8–12 children was moderately associated to hypertension. Many children may be a source of sleepless nights to parents especially fathers. Abnormal sleep upsets endocrine and metabolic performance and increases the sympathetic nervous system activity leading to increased risk of hypertension [45].

We observed no association between low physical activity, fruit and vegetable intake per week, work status in the past 12 months, frequently added salt to food at the table and frequently added sugar to tea/coffee with hypertension despite previous reports on association [1, 16, 17, 46, 47]. As a culinary tradition, Fulani, especially men, consume highly salted roasted meat and sugar-laden local tea throughout the whole day. Meat intake, especially red meat, dietary sodium and sugar intake increases blood pressure and risk of hypertension [9, 47]. This may account for high prevalence of grades 1, 2 and 3 hypertension and high mean values of blood pressure in men.

This may be due to the challenges involved with population-based studies or subjectivity in the measure and classification of these risk factors. Unlike in our study, low physical activity has been associated with hypertension [48, 49]. Cigarette smoking was associated with hypertension as shown by previous studies. Smoking is a modifiable risk factor for hypertension and important in intervention programs [50, 51].

Hypertension is a constellation of pathogenesis and pathophysiology causes involving lifestyle and environmental factors, genetic predisposition, disruption in the vasculature and neurohumoral synergy. Hypertension is associated with metabolic dysfunction and multisystem structural impairment which facilitate cardiovascular risk. Hypertension alone is the most important risk factor for stroke and a major factor of cognitive decline in later life.

Rural poverty, environmental degradation, forced migrations and limited access to health have combined to create and perpetuate deleterious health consequences for aboriginal people from hypertension, diabetes, obesity, cardiovascular disease, interpersonal violence and suicide, attributed to the increasing use of alcohol and drugs [52–54]. Hypertensive individuals as compared to normotensive individuals have a twofold increased risk of developing coronary artery disease, fourfold increased risk of congestive heart failure and sevenfold increased risk of cerebrovascular and stroke [55]. This is exacerbated by unresponsive and under-resourced health systems and limited research among indigenous populations [56]. An upsurge in morbidity and mortality from hypertension may be witnessed in the near future among the Fulani population. We are not aware of a previous study that highlighted the burden of hypertension in the Fulani, majority of whom are indigenous Mbororo. As more Fulani move to urban centres and embrace “westernised” lifestyle, the prevalence of hypertension in this population may increase. Future strategies to tackle hypertension should focus on culturally embedded prevention, delay of the onset and the evolution of the silent disease process among the Fulani.

## Conclusion

The study provided data on occurrence and drivers of hypertension among the Fulani as they undergo transition from traditional to transitory and modern lifestyles. Our findings indicate a high prevalence of hypertension in the rural Fulani populations, low awareness and control. Rural settings and life may not be protective for hypertension for this population. The results of this study are critical for improving hypertension control and reducing CVD in this population with low healthcare accessibility, under-resourced healthcare system, low education, health care beliefs and practices at variance with the general population. The Fulani populations should benefit from early detection and culturally embedded appropriate interventions.

### Study strengths and limitations

Strengths of the study included a large sample size of the Fulani population, the majority of whom are indigenous Mbororo population with no extant knowledge on NCD health risks. We used standard conditions and automated devices to carry out the study in order to reduce measurement errors and bias that may arise from training multiple research teams. Our researchers were conversant with local Fulani language which was often used to administer questionnaires. BMI reliability in screening for cardio-metabolic abnormalities could miss many at-risk adults in some racial/ethnic groups but in this study, we combined BMI-defined overweight/obesity and WC in assessing hypertension risk. Further, we used risk factor analysis centred on individuals as a preponderant scale in probing epidemiological questions and adequately explaining who is at risk. WHO and the 2013 ESH/ESC guidelines for definition and classification of hypertension were used. The international definition of BMI subclasses, IR/SIR from WC was used. Limited research is done on the health of indigenous populations [56], and the study enriched the knowledge base of hypertension in Fulani indigenous populations. Evidence from this study provides an opportunity for active surveillance, diagnosis, treatment of patients and capacity building as we noted low awareness of hypertension in this population. The results allow for valuable ethnic-specific hypotheses and are vital for further research.

An important limitation of this study is the observational, cross-sectional design which precludes examination of multiple scale causal mechanisms and findings to allow for causal deductions. Mention should be made of unmeasured variables that may lead to residual confounding and measured confounders that can lead to misclassification as one of the limitations. Also, age-related associations to hypertension may also be due to lifestyle differences across age groups and not age trends. The elderly were also transitioning from traditional to transitional and modern ways of life as well as the young. This population though described as rural does not live in isolation. They interact regularly with neighbouring urban populations in towns and are exposed to urban or semi-urban life and could equally be considered as semi-urban. Risk factor analysis does not explain why risks exist or differ within and between populations. Longitudinal studies are required to confirm the results. Risk factors are a continuum, and categorisation disregards considerable contributions brought by these factors below threshold levels. There are challenges to risk factor analysis due to the complexity of fundamental causes of diseases such as social, biological, behavioural and economic drivers, poor hygiene, infections, overcrowding and gene interactions with environments and life course trajectories associated with poor health amongst indigenous populations [54]. Seasonal variations of blood pressure, effect modification from socio-economic status and indoor temperature changes on blood pressure were not assessed and might have influenced blood pressure readings and hypertension [57]. The study did not differentiate Fulani/Peul people into Fulbe (settled Fulani) and Mbororo (nomadic pastoral Fulani) [10] who may have different epidemiological profiles though sharing the same ancestry. Our findings may not be generalizable to the larger population of Fulani in some countries, the majority of whom are nomadic with different disease and risk factor burdens moderated by contextual political, socio-economic and health systems. Fruit and vegetable quantification did not take into consideration servings eaten per day. No detailed exploration on sleep difficulty was done.

## Additional files

Additional file 1: Table S1. Multivariable logistic regression for Fulani men and women (adjusted for site) and both men and women (adjusted for site and gender). (DOCX 55 kb)

Additional file 2: Table S2. Univariate analysis by gender for Fulani aged ≥ 20 years, 2013. (DOCX 75 kb)
