# Supplementary material for: Epidemiology of hypertension in Fulani indigenous populations—age, gender and drivers
Source: J Health Popul Nutr. 2017 Nov 10;36:35. doi: 10.1186/s41043-017-0112-2 (PMC5681807; doi:10.1186/s41043-017-0112-2)
Supplement: Supplementary file 1 — Multivariable logistic regression for Fulani men and women (adjusted for site) and both men and women (adjusted for site and gender). (DOCX 55 kb) [file 41043_2017_112_MOESM1_ESM.docx]

Table: Multivariable logistic regression for Fulani men & women (adjusted for site) and both men & women (adjusted for site and gender)

| **Models** | **Men, 425 (Model 1)** | | | **Women, 912 (Model 2)** | | |
| --- | --- | --- | --- | --- | --- | --- |
| **Factors** | **AOR** | **95% CI** | **p-value** | **AOR** | **95% CI** | **p-value** |
| Site | 1.11 | 0.91-1.34 | 0.298 | 0.97 | 0.83-1.13 | 0.689 |
| **Age group** |  |  |  |  |  |  |
| 20-39 | **Ref** |  |  | **Ref** |  |  |
| 40-59 | **1.41** | **1.05-2.45** | **0.033** | **3.04** | **2.05-4.51** | **<0.001** |
| ≥60 | **3.65** | **1.94-6.86** | **<0.001** | **7.91** | **3.90-16.02** | **<0.001** |
| **Marital status** |  |  |  |  |  |  |
| Single | Ref |  |  | Ref |  |  |
| Presently married | 1.48 | 0.65-3.39 | 0.353 | 1.01 | 0.41-2.48 | 0.987 |
| Divorced/Separated | 2.06 | 0.64-6.69 | 0.227 | 1.45 | 0.54-3.91 | 0.463 |
| **Level of Education** |  |  |  |  |  |  |
| Attended school | **Ref** |  |  | Ref |  |  |
| Never attended school | **1.61** | **1.01-2.64** | **0.045** | 1.27 | 0.82-1.96 | 0.289 |
| **Smoking habit** |  |  |  |  |  |  |
| Abstainer |  |  |  | Ref |  |  |
| Current/Former |  |  |  | ***3.34*** | ***0.97-11.45*** | ***0.055*** |
| **Diabetes** |  |  |  |  |  |  |
| No | **Ref** |  |  | **Ref** |  |  |
| Yes | **6.39** | **2.35-17.38** | **0.001** | ***2.25*** | ***0.98-5.16*** | ***0.055*** |
| **FH of hypertension** |  |  |  |  |  |  |
| No |  |  |  | **Ref** |  |  |
| Yes |  |  |  | **1.74** | **1.15-2.63** | **0.009** |
| **Number of children** |  |  |  |  |  |  |
| Non | Ref |  |  | Ref |  |  |
| 1 – 2 children | 1.74 | 0.63-4.82 | 0.282 | 0.81 | 0.37-1.76 | 0.598 |
| 3 – 4 children | 1.07 | 0.39-2.95 | 0.889 | 0.71 | 0.33-1.53 | 0.376 |
| 5 – 7 children | 1.46 | 0.53-4.06 | 0.463 | 0.66 | 0.29-1.47 | 0.308 |
| 8 – 12 children | ***2.53*** | ***0.95-6.72*** | ***0.063*** | 0.92 | 0.39-2.19 | 0.857 |
| ≥13 children | 1.85 | 0.76-4.49 | 0.175 | 0.97 | 0.41-2.28 | 0.949 |
| **Sleeping difficulties?** |  |  |  |  |  |  |
| Sleep normally | **Ref** |  |  |  |  |  |
| Have insomnia | **1.63** | **1.02-2.62** | **0.042** |  |  |  |
| **BMI** |  |  |  |  |  |  |
| Normal weight |  |  |  | Ref |  |  |
| Under weight |  |  |  | 0.79 | 0.56-1.14 | 0.220 |
| Overweight |  |  |  | 1.54 | 0.81-2.91 | 0.184 |
| Obese |  |  |  | 1.43 | 0.60-3.38 | 0.419 |
| **WC** |  |  |  | **MBF** |  |  |
| LR (≤94M or ≤80W)cm | **Ref** |  |  | **Ref** |  |  |
| IR(94>&<102M or 80>&<88W)cm | ***3.07*** | ***0.96-9.85*** | ***0.058*** | 0.81 | 0.52-1.29 | 0.380 |
| SIR (≥102M or ≥88 W) cm | ***5.59*** | ***0.88-35.63*** | ***0.068*** | **1.58** | **1.01-2.45** | **0.043** |
