# Supplementary material for: Epidemiology of hypertension in Fulani indigenous populations—age, gender and drivers
Source: J Health Popul Nutr. 2017 Nov 10;36:35. doi: 10.1186/s41043-017-0112-2 (PMC5681807; doi:10.1186/s41043-017-0112-2)
Supplement: Supplementary file 2 — Univariate analysis by gender for Fulani aged ≥ 20 years, 2013. (DOCX 75 kb) [file 41043_2017_112_MOESM2_ESM.docx]

| Table: Univariate analysis by gender for Fulani aged ≥20 years, 2013 | | | | | | | | | |
| --- | --- | --- | --- | --- | --- | --- | --- | --- | --- |
|  | **Fulani men, 425** | | | **Fulani women, 912** | | | **Both Fulani men and women, 1 337** | | |
| **Factors** | OR | 95% CI | p-value | OR | 95% CI | p-value | OR | 95% CI | p-value |
| **Site** | 1.13 | 0.96-1.33 | 0.144 | 0.99 | 0.87-1.15 | 0.998 | 1.03 | 0.93-1.15 | 0.587 |
| **Gender** |  |  |  |  |  |  |  |  |  |
| Women |  |  |  |  |  |  | **Ref** |  |  |
| Men |  |  |  |  |  |  | **1.42** | **1.12-1.81** | **0.004** |
| **Age group** |  |  |  |  |  |  |  |  |  |
| 20-39 | Ref |  |  | Ref |  |  | **Ref** |  |  |
| 40-59 | **2.37** | **1.48-3.79** | **<0.001** | **3.56** | **2.54-4.99** | **<0.001** | **3.15** | **2.41-4.13** | **<0.001** |
| ≥60 | **4.96** | **2.73-8.99** | **<0.001** | **11.08** | **6.04-20.33** | **<0.001** | **7.86** | **5.25-11.77** | **<0.001** |
| **Marital status** |  |  |  |  |  |  |  |  |  |
| Single | **Ref** |  |  | **Ref** |  |  | **Ref** |  |  |
| Presently married | **2.74** | **1.46-5.12** | **0.002** | 0.94 | 0.41-2.16 | 0.876 | 1.56 | 0.95-2.54 | 0.077 |
| Divorced/Separated | **5.92** | **2.06-17.02** | **0.001** | **2.75** | **1.11-6.78** | **0.028** | **3.87** | **2.16-6.95** | **<0.001** |
| **Education level** |  |  |  |  |  |  |  |  |  |
| Attended school | **Ref** |  |  | Ref |  |  | **Ref** |  |  |
| Never | **2.03** | **1.33-3.09** | **0.001** | **1.58** | **1.04-2.39** | **0.031** | **1.59** | **1.19-2.11** | **0.001** |
| **Currently employed** |  |  |  |  |  |  |  |  |  |
| No | Ref |  |  | Ref |  |  | Ref |  |  |
| Yes | 1.17 | 0.64-2.13 | 0.605 | 0.81 | 0.55-1.18 | 0.264 | 0.92 | 0.67-1.26 | 0.589 |
| **Smoking habit** |  |  |  |  |  |  |  |  |  |
| Abstainer | Ref |  |  | **Ref** |  |  | **Ref** |  |  |
| Former/current smoker | 1.43 | 0.87-2.35 | 0.162 | **7.83** | **2.79-21.9** | **<0.001** | **2.36** | **1.58-3.54** | **<0.001** |
| **Alcohol drinking** |  |  |  |  |  |  |  |  |  |
| Never | Ref |  |  | Ref |  |  | **Ref** |  |  |
| Occasional/daily drinkers | 1.48 | 0.85-2.59 | 0.165 | 1.51 | 0.71-3.21 | 0.281 | **1.66** | **1.08-2.55** | **0.021** |
| **Vegetable intake** |  |  |  |  |  |  |  |  |  |
| 6 – 7 days / week | Ref |  |  | Ref |  |  | Ref |  |  |
| 3 – 5 days / week | 1.02 | 0.66-1.56 | 0.935 | 1.06 | 0.77-1.47 | 0.705 | 1.05 | 0.81-1.36 | 0.687 |
| 0 – 2 days / week | 0.99 | 0.53-1.83 | 0.974 | 1.06 | 0.67-1.67 | 0.805 | 1.04 | 0.71-1.52 | 0.836 |
| **Fruit intake** |  |  |  |  |  |  |  |  |  |
| 6 – 7 days / week | Ref |  |  | Ref |  |  | Ref |  |  |
| 3 – 5 days / week | 1.42 | 0.76-2.65 | 0.276 | 1.10 | 0.69-1.75 | 0.672 | 1.22 | 0.84-1.76 | 0.292 |
| 0 – 2 days / week | 1.39 | 0.77-2.53 | 0.275 | 1.23 | 0.80-1.88 | 0.433 | 1.28 | 0.91-1.79 | 0.162 |
| **Physical activity** |  |  |  |  |  |  |  |  |  |
| Vigorous | Ref |  |  | Ref |  |  | **Ref** |  |  |
| Moderate | 0.97 | 0.59-1.59 | 0.915 | 0.83 | 0.58-1.18 | 0.300 | 0.84 | 0.63-1.12 | 0.237 |
| Low | 1.37 | 0.85-2.22 | 0.193 | 1.07 | 0.75-1.52 | 0.718 | 1.12 | 0.84-1.49 | 0.432 |
| **10 minutes leisure PA** |  |  |  |  |  |  |  |  |  |
| Vigorous | Ref |  |  | Ref |  |  | Ref |  |  |
| Moderate | 0.83 | 0.27-2.49 | 0.738 | 0.64 | 0.24-1.76 | 0.390 | 0.70 | 0.34-1.47 | 0.350 |
| Low | 1.07 | 0.66-1.74 | 0.771 | 0.79 | 0.53-1.19 | 0.272 | 0.86 | 0.64-1.16 | 0.326 |
| **Always added salt** |  |  |  |  |  |  |  |  |  |
| No | Ref |  |  | Ref |  |  |  |  |  |
| Yes | 0.96 | 0.55-1.64 | 0.872 | 0.81 | 0.55-1.20 | 0.294 | 0.85 | 0.62-1.17 | 0.325 |
| **Always added sugar** |  |  |  |  |  |  |  |  |  |
| No | Ref |  |  | Ref |  |  | Ref |  |  |
| Yes | 0.86 | 0.52-1.41 | 0.545 | 0.97 | 0.66-1.43 | 0.893 | 0.95 | 0.71-1.28 | 0.752 |
| **FH of hypertension** |  |  |  |  |  |  |  |  |  |
| No | Ref |  |  | **Ref** |  |  | **Ref** |  |  |
| Yes | 1.57 | 0.89-2.74 | 0.112 | **1.77** | **1.22-2.58** | **0.003** | **1.69** | **1.25-2.29** | **0.001** |
| **FH of diabetes** |  |  |  |  |  |  |  |  |  |
| No | Ref |  |  | Ref |  |  | Ref |  |  |
| Yes | 1.71 | 0.83-3.54 | 0.146 | 1.32 | 0.81-2.16 | 0.266 | 1.42 | 0.94-2.13 | 0.094 |
| **FH of obesity** |  |  |  |  |  |  |  |  |  |
| No | Ref |  |  | Ref |  |  | Ref |  |  |
| Yes | 1.51 | 0.49-4.61 | 0.465 | 1.35 | 0.69-2.65 | 0.374 | 1.35 | 0.77-2.37 | 0.289 |
| **Diabetes** |  |  |  |  |  |  |  |  |  |
| No | **Ref** |  |  | **Ref** |  |  | **Ref** |  |  |
| Yes | **7.12** | **2.34-21.69** | **0.001** | **2.75** | **1.36-5.54** | **0.005** | **3.68** | **2.07-6.53** | **<0.001** |
| **Sleeping difficulties?** |  |  |  |  |  |  |  |  |  |
| Sleep normally | **Ref** |  |  | Ref |  |  | **Ref** |  |  |
| Have insomnia | **1.73** | **1.09-2.73** | **0.019** | 1.31 | 0.93-1.86 | 0.124 | **1.44** | **1.09-1.91** | **0.010** |
| **Planning to migrate?** |  |  |  |  |  |  |  |  |  |
| Yes | Ref |  |  | Ref |  |  | Ref |  |  |
| No | 1.69 | 0.86-3.37 | 0.130 | 0.93 | 0.52-1.67 | 0.804 | 1.14 | 0.74-1.76 | 0.550 |
| Don’t know | 1.16 | 0.46-2.95 | 0.741 | 1.32 | 0.64-2.69 | 0.451 | 1.27 | 0.73-2.23 | 0.393 |
| **Number of children** |  |  |  |  |  |  |  |  |  |
| Non | **Ref** |  |  | **Ref** |  |  | **Ref** |  |  |
| 1 – 2 children | 1.83 | 0.76-4.41 | 0.179 | 0.71 | 0.33-1.49 | 0.364 | 0.99 | 0.58-1.69 | 0.978 |
| 3 – 4 children | 1.76 | 0.76-4.09 | 0.188 | 0.83 | 0.39-1.75 | 0.616 | 1.13 | 0.66-1.92 | 0.664 |
| 5 – 7 children | **2.64** | **1.12-6.23** | **0.027** | 1.08 | 0.51-2.32 | 0.835 | 1.50 | 0.88-2.56 | 0.132 |
| 8 – 12 children | **4.89** | **2.17-11.03** | **<0.001** | **2.41** | **1.11-5.22** | **0.027** | **3.37** | **1.95-5.83** | **<0.001** |
| ≥13 children | **2.42** | **1.09-5.37** | **0.030** | 1.09 | 0.48-2.53 | 0.824 | 1.62 | 0.93-2.84 | 0.089 |
| **BMI** |  |  |  |  |  |  |  |  |  |
| Normal weight | Ref |  |  | **Ref** |  |  | **Ref** |  |  |
| Under weight | 0.71 | 0.47-1.07 | 0.101 | 0.80 | 0.58-1.11 | 0.188 | **0.74** | **0.57-0.96** | **0.023** |
| Overweight | 0.55 | 0.17-1.84 | 0.333 | **1.91** | **1.06-3.44** | **0.030** | 1.39 | 0.84-2.32 | 0.199 |
| Obese | 3.81 | 0.71-20.26 | 0.117 | 1.84 | 0.76-4.44 | 0.174 | 2.00 | 0.96-4.17 | 0.062 |
| **WC** |  |  |  |  |  |  |  |  |  |
| LR (≤94M or ≤80W)cm | **Ref** |  |  | **Ref** |  |  | **Ref** |  |  |
| IR(94>&<102M or 80>&<88W)cm | **5.74** | **1.80-18.29** | **0.003** | 0.98 | 0.63-1.51 | 0.919 | 1.07 | 0.73-1.56 | 0.732 |
| SIR (≥102M or ≥88 W)cm | ***4.78*** | ***0.91-25.17*** | ***0.065*** | **1.96** | **1.29-2.98** | **0.002** | **1.82** | **1.23-2.67** | **0.003** |
